# Influence of Methacrylate and Vinyl Monomers on Radical Bulk Photopolymerization Process and Properties of Epoxy-Acrylate Structural Adhesives

**DOI:** 10.3390/polym15040926

**Published:** 2023-02-13

**Authors:** Konrad Gziut, Agnieszka Kowalczyk, Beata Schmidt, Tomasz J. Idzik, Jacek G. Sośnicki

**Affiliations:** 1 Department of Chemical Organic Technology and Polymeric Materials, Faculty of Chemical Technology and Engineering, West Pomeranian University of Technology in Szczecin, 70-322 Szczecin, Poland; 2 Department of Organic and Physical Chemistry, Faculty of Chemical Technology and Engineering, West Pomeranian University of Technology in Szczecin, 71-065 Szczecin, Poland

**Keywords:** epoxy acrylates, polymer synthesis, photopolymerization, structural adhesives, adhesion, thermal analysis

## Abstract

In this paper, epoxy-acrylate structural adhesives tapes (SATs) were obtained from Bisphenol A-based liquid epoxy resin and epoxy acrylic resins (EARs). A new method of EARs preparation, i.e., the free radical bulk photopolymerization process (FRBP), was studied in detail. The influence of methacrylic monomers (methyl methacrylate, ethyl methacrylate, butyl methacrylate, lauryl methacrylate, (2-acetoacetoxy)ethyl methacrylate) and vinyl monomers (N-vinylpyrrolidone and styrene) on the FRBP process of base monomers (i.e., butyl acrylate, glycidyl methacrylate and 2-hydroxyethyl acrylate) was investigated. The kinetics of photopolymerization process was monitored by photo-differential scanning calorimetry method. The properties of the obtained EARs (viscosity and average molecular weights), as well as monomers conversion using ^1^H NMR, were determined. It was revealed that styrene significantly decreases the photopolymerization rate and increases the final monomers conversion (+27%). However, the resulting tetrapolymers BA-co-GMA-co-HEA-co-STY have low molecular weights and low polydispersity (2.2). Methacrylate monomers with shorter aliphatic chains (<C_4_) also decrease the rate of photopolymerization due to the length of the aliphatic chain increasing. Surprisingly, the best results of adhesion to steel and shear strength were obtained for SAT based on epoxy acrylate resin with styrene (11 N/25 mm and 20.8 MPa, respectively). However, the thermomechanical properties of SAT with styrene were weaker than those with methacrylates.

## 1. Introduction

Epoxy functional acrylics, referred to as epoxy acrylates (EA), are defined as acrylic copolymers carrying epoxy groups. The EA polymers are synthesized in a free radical polymerization reaction of monomers containing a vinyl or acrylic/methacrylic double bond. The most used monomer is glycidyl methacrylate (GMA). The standard synthesis method for EA is solution polymerization in semi-batch process, mass polymerization at high temperature (ca. 230 °C), suspension polymerization or polymerization in supercritical carbon dioxide [1]. EA polymers can be classified as special types of acrylic resins (ARs) that are widely used to produce coatings, adhesives, printing inks, dental materials [2,3,4,5,6,7] or in 3D printing [8,9]. The popularity of ARs is evidenced by the value of the global market (16.8 billion in 2020 and is expected to reach 21.9 billion in 2025) [10,11]. In the beginning, the acrylic resins used in the coating materials were obtained as 30–40% high molecular weight solutions. This changed in the early 20th century due to volatile organic content (VOC) requirements that became more restrictive because of increasing awareness of environmental protection [12]. Countries around the world have formulated laws to limit the emission of VOCs. This has limited the development of traditional solvent-based coatings. As a result, formulations with a lower solvent content, and thus a high solid content (70—80%), have become more typical [13].

To meet the global requirements and produce resins in a more sustainable way and with less VOCs, scientists are developing various methods of obtaining ARs. One such method is to use biobased acrylic monomers in place of conventional monomers which are currentlyproduced primarily from crude oil [14]. Another is the replacement of organic solvents with water. However, waterborne acrylic resins must be properly modified to eliminate their main drawbacks, like low solids content and poor water and corrosion resistance [15]. One interesting approach seems to be the synthesis of ARs by the free-radical bulk photopolymerization process (FRBP), which is a new approach in mass polymerization. Such a method does not only allow the exclusion of organic solvents but also saves time and energy (it does not require an elevated temperature to decompose the initiator), especially since it can be carried out using conventional LEDs [16]. FRBP allows products called polymer syrups (PSs) to be obtained, which are equivalent to polymer resins. However, PSs contain unreacted monomers, rather than an organic solvent. PSs can be used as binders of adhesives or varnishes [17,18]. Nevertheless, in publications that describe attempts to obtain acrylic syrups by bulk photopolymerization, the conversion of acrylic monomers to polymers is usually less than 20% [19,20]. Achieving higher conversions (>50% or 70%) while maintaining a useful viscosity (<50 Pa∙s) seems to be of key importance for the obtained products to share properties with known acrylic resins and to be environmentally friendly.

The difficulty in achieving high monomers conversions is because the photopolymerization process is carried out in bulk (to exclude organic solvents) and is initiated at great speed using light, which causes an immediate increase in temperature and limited control over chains growth. It should be noted that, during radical polymerization, uncontrolled chain branching and autoacceleration can very easily occur, leading to very high viscosities and renders the product useless [21,22]. It is also known that the viscosity of the systems increases with increasing molecular weight of the polymer. Thus, to obtain resins with higher polymer content (solid content) and not too high viscosity, lower molecular weight polymers are obtained. For example, this can be achieved by increasing the amount of initiator (up to 6% in classic, thermal polymerization) with or without using chain transfer agents [23]. The photoinitiator, however, is usually the most expensive component of the photopolymerizable composition. Moreover, in the case of immediate light initiation, the use of a large amount of photoinitiator can easily lead to the formation of a gel fraction and to yellow product, as the photobleaching will not be completed before the end of the process [24].

In previous articles, the influence of the photoinitiator and the agitation rate of the reactor feed on the FRBP process were investigated [16,24]. Bearing in mind that, in the case of bulk photopolymerization, nearly 100% of the reactor feed consists of monomer(s), it seems important to check the influence of the comonomer type on the course of the process, achieved conversions and average molecular weights of the products. In the case of thin-layer photopolymerization, a few monomers that slow down the radical polymerization process of acrylates have already been described i.e., methyl methacrylate and styrene [25]. Their effect was considered unfavorable due to the prolonged reaction time and the reduction of the maximum conversion [26]. However, it seems that this behavior may be desirable in the case of FRBP process as the reaction may proceed in a more controlled manner, allowing us to obtain resins with higher monomers conversions.

The goal of this work is to understand the influence of selected and commonly used methacrylic and vinyl monomers on the course of the photopolymerization process carried out in the bulk (without solvents) during mechanical mixing of the comonomers, as well as the physico-chemical properties of obtained epoxy acrylic resins (EARs). Moreover, prepared EARs (as adhesive binders) were mixed with epoxy resins to obtain epoxy-acrylate structural adhesives tapes (SATs). These adhesives are characterized by relatively high adhesion to a metal or ceramic substrate, as well as high tack. They behave like typical pressure-sensitive adhesives. However, after thermal curing, they exhibit high shear strength like typical structural adhesives (e.g., liquid one-component epoxy adhesives). However, the shear strength should be at least 7 MPa. In this paper, the effect of methacrylic and vinyl monomers in the structure of adhesive binders (EARs) was studied to determine the thermal and mechanical properties of SATs.

## 2. Materials and Methods

### 2.1. Materials

The following components were used to prepare epoxy acrylic resins (EARs) via the bulk photopolymerization process:

(I) base monomers: n-butyl acrylate (BA), glycidyl methacrylate (GMA), 2-hydroxyethyl acrylate (HEA) (Merck Group, Warsaw, Poland);
(II) methacrylic monomers: methyl methacrylate (MMA), ethyl methacrylate (EMA), butyl methacrylate (BMA), lauryl methacrylate (LMA), (2-acetoacetoxy)ethyl methacrylate (AEM) (Merck Group, Warsaw, Poland)
(III) vinyl monomers: N-vinylpyrrolidone (NVP) (BASF, Ludwigshafen, Germany) and styrene (STY) (Merck Group, Warsaw, Poland),

The chemical structures of the comonomers are shown in Table 1. As a radical photoinitiator, an acylphosphine oxide type photoinitiator was used, i.e., 2,4,6-trimethylbenzoyl-diphenyl phosphine oxide (Omnirad TPO, IGM Resins, Waalwijk, The Netherlands).

Epoxy-acrylate structural adhesive tapes (SATs) were compounded using EARs and the Bisphenol A-based liquid epoxy resin with an epoxy equivalent weight of 202 g/equiv. and viscosity of 25 Pa∙s (Epidian; Ciech Sarzyna, Nowa Sarzyna, Poland), radical photoinitiator Omnirad 127 (IGM Resin, The Netherlands), the multifunctional acrylic monomer (Laromer 9023; BASF, Ludwigshafen, Germany), the Lewis acid adduct (as a latent curing agent) (Nacure Super Catalyst A218; Worleé Chemie, Hamburg, Germany), and an adhesion promoter (Byk 4510 (Byk-Chemie, Wesel, Germany).

### 2.2. Synthesis of Epoxy Acrylic Resins

**Figure 1:**
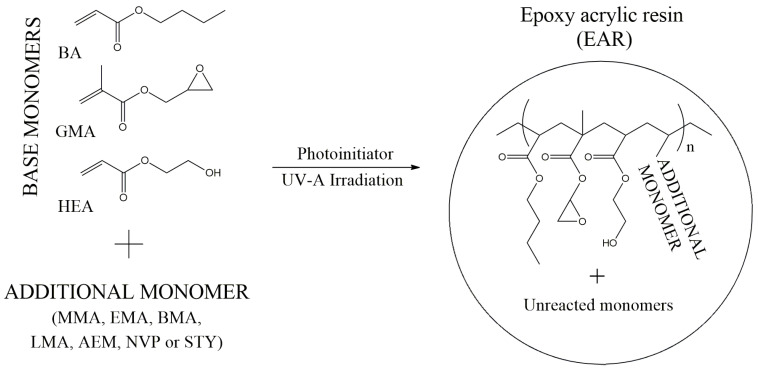
Preparation of epoxy acrylic resins via free-radical photopolymerization process.

The epoxy acrylic resins (EARs) were prepared via free-radical bulk-photopolymerization process (FRBP) conducted in glass reactor (250 mL) equipped with a mechanical stirrer, thermocouple, and inert gas supply. The reaction mixture (50 g) was introduced into the reactor and purged with argon for 20 min. The mixtures consisted of three base monomers (BA, GMA, HEA), a photoinitiator (TPO) and an additional comonomer (MMA, EMA, BMA, LMA, AEM, NVP or STY) was introduced. Each of the base monomers is extremely important in the preparation of structural adhesive tapes (SATs), which have self-adhesive properties (before the thermal curing process) and structural mechanical strength (after thermal curing). Namely, BA lowers the *T_g_* value of adhesive (self-adhesive properties), while GMA and HEA take part in the cationic polymerization of epoxy groups from epoxy resin (polymer network formation). The additional comonomer (like methacrylic or vinyl monomer) was introduced to increase control over the FRBP process. The schematic structure of tetrapolymer chains is shown in Figure 1.

Copolymerizations were realized at an initial temperature of 21 ± 2 °C, controlled with water-cooling. The temperature of the reaction mixture was monitored during the synthesis. Each process was terminated (irradiation stopped) 10 min after the recorded temperature peak. The amount of photoinitiator and the reaction time were selected experimentally in such a way as to obtain an EARs with the highest monomers conversion possible for a given system, assuming that no gel fractions will be formed and the viscosity will remain at a level allowing for further use of the product (approx. 10 Pa∙s). The source of the UV radiation was a UV-LED stripe (MEiSSA, Warsaw, Poland) with a wavelength of 390 nm ± 5 nm. The intensity of the UV radiation inside the reactor, measured with a UV-radiometer SL2W (UV-Design, Germany), was 10 mW/cm^2^. The compositions of the reaction mixtures are presented in Table 2.

The kinetics of the UV-induced polymerization process were monitored with photo-differential scanning calorimeter with a UV attachment (DSC Q100, TA Instruments, New Castle, DE, USA; UV-light emitter Omnicure S2000; Excelitas Technologies, Malvern, PA, USA) at room temperature (isothermal measurement). Samples (ca. 5 mg) were irradiated with UV in the range of 320–390 nm, with an intensity of 10 mW/cm^2^ in argon atmosphere. Photo-DSC experiments were conducted in triplicate. The photopolymerization rate (*R_p_*, s^−1^) was calculated according to Equation (1), and the conversion of double bonds (*p*, %)—according to Equation (2).

(1) $$R_{p}=\frac{\left(\frac{dH}{dt}\right)}{H_{0}}\left[s^{-1}\right]$$

(2) $$p=\frac{\Delta H_{t}}{\Delta H_{0}}\cdot 100\;\left[\%\right]$$

where: *dH*/*dt* is the heat flow recorded during UV-irradiation, H_0_ is the theoretical heat value for the complete degree of conversion (∆*H* = 78.0 kJ/mol for acrylates, ∆*H* = 54.0 kJ/mol for methacrylates, ∆*H* = 67.4 kJ/mol for STY, ∆*H* = 53.9 kJ/mol for NVP), ∆*H_t_* is the reaction heat evolved at time *t*.

### 2.3. Characterization of the Epoxy Acrylic Resins

Dynamic viscosity of the EARs was measured at 21 °C by means of DV-II Pro Extra viscometer (Brookfield, New York, NY, USA). Solids content (SC) was determined using Moisture Analyzer MA 50/1.X2.IC.A (Radwag, Radom, Poland). Samples (ca. 1 g) were heated in aluminum scale pans at a temperature of 105 °C for 4 h. Monomers conversion was determined by proton nuclear magnetic resonance ^1^H NMR (Bruker DPX Avance III HD Spectrometer; 400 MHz). Naphthalene as internal standard was used and samples of ARs were dissolved in CDCl_3_. The conversion of monomers was determined by comparing the intensity of monomer peaks against the intensity of peaks of the internal standard at 7.5 ppm and 7.8 ppm (naphthalene).

Average molecular weights (Mw, Mn) and polydispersity (PDI) were determined by gel permeation chromatography (GPC). The GPC apparatus contained the refractive index detector (Merck Lachrom RI L-7490), pump (Merck Hitachi Liquid Chromatography L-7100), interface (Merck Hitachi Liquid Chromatography D-7000) and the Shodex OHpak SB-806M MQ column with Shodex OHpak SB-G precolumn. The GPC tests were performed using polystyrene standards (Fluka and Polymer Standards Service GmbH, Mainz, Germany) and tetrahydrofurane. Prior to testing, the EARs were dried at 110 °C for 1 h to remove unreacted monomers.

### 2.4. Preparation and Characterization of Structural Adhesive Tapes (SATs) and Al/SAT/Al Joints

**Figure 2:**
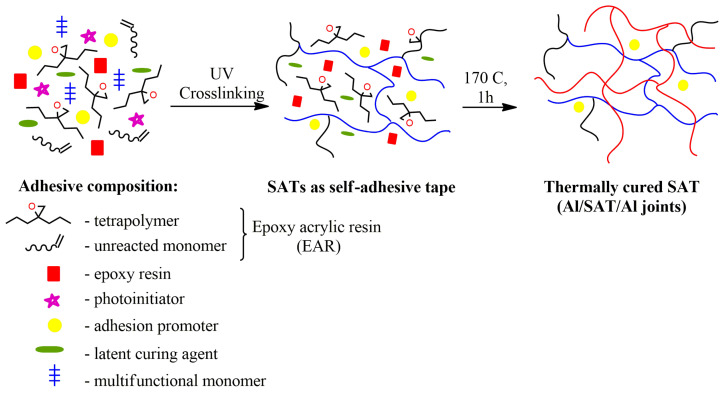
Preparation of SATs and Al/SAT/Al joints.

Adhesive compositions were prepared by compounding the epoxy resin (50 wt.%) with the obtained EAR (50 wt.%) as an adhesive binder. A latent curing agent (1.5 wt. parts), multifunctional monomer (2 wt. parts), photoinitiator (Omnirad 127; 2 wt. parts) and adhesion promoter (0.75 wt. part) were then incorporated into the adhesive binders. After mechanical mixing, they formed stable, homogeneous mixtures (adhesive compositions). The addition of a multifunctional monomer increases the cross-linking density of the polyacrylate network, formed during the subsequent UV cross-linking. The preparation of SATs is schematically shown in Figure 2.

The prepared SATs compositions were applied onto polyester foils (samples for self-adhesive tests) or siliconized paper (other tests) by adjustable micrometer film applicator. The obtained thin layers of the composition were subjected to UV radiation (2—4 J/cm^2^) using a medium pressure mercury lamp (UV-ABC; Hönle UV-Technology, Gräfelfing, Germany). The UV irradiation was controlled with the radiometer (Dynachem 500; Dynachem Corp., Westville, Il, USA). The thickness of the UV-crosslinked SATs layers were 120 ± 10 µm. The self-adhesive properties of the thermally uncured SATs were tested according to AFERA 5001 (adhesion to a steel substrate) and AFERA 5015 (tack). These parameters were evaluated using three samples of each adhesive tape by means of the Z010 machine (Zwick/Roell, Ulm, Germany).

The glass transition temperatures (*T_g_*) of thermally uncured SATs and enthalpy of curing processes (Δ*H*) were determined using differential scanning calorimetry (DSC Q100, TA Instruments, New Castle, DE, USA). Each analysis was carried out using hermetic aluminum pans in the temperature range from —80 to 350 °C at a heating rate of 10 °C/min.

SAT films (after UV-crosslinking process) and degreased 2024 aluminum panels (100 × 25 × 1.6 mm) were used to construct Aluminum–SAT–Aluminum overlap joints (Al/SAT/Al). The joints were thermally cured at 170 °C for 60 min and then their shear strength was measured at room temperature according to the ASTM D1002-10 standard using Instron 5982 machine (Instron, Norwood, MA, USA). The cross-linking degree (*α*) of thermally cured SATs was calculated using DSC data according to Equation (3) [27].

(3) $$\alpha =\left(\frac{\Delta H_{T}-\Delta H_{res}}{\Delta H_{T}}\right)\;\left(a.u.\right)$$

where: Δ*H_T_* is the total enthalpy of the SAT curing process (J/g) and Δ*H_res_* is the enthalpy of a postcuring process of the thermally cured SAT (in an Al/SAT/Al joint).

Dynamic mechanical analysis (DMA) was performed by using the DMA Q800 (TA Instruments, USA). The testing configuration was the dual cantilever mode, with the nominal sample dimension of 50 × 10 × 2 mm and a heating ramp of 3 °C/min. All tests were performed by setting 20 μm as the amplitude and 1 Hz as the frequency. For thermal (DSC) and thermo-mechanical (DMA) properties, three tests for each sample were performed.

## 3. Results

### 3.1. Kinetic Study of Photopolymerization Process

**Figure 3:**
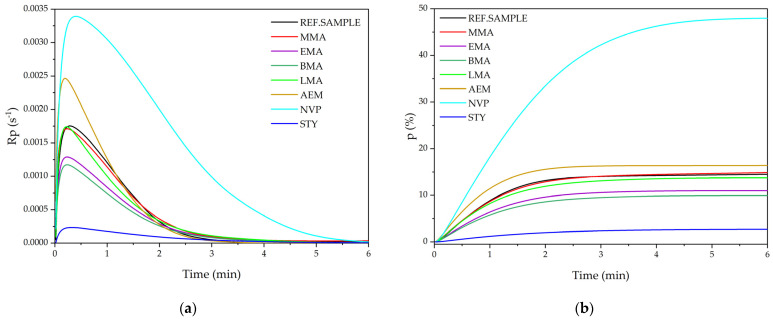
The photopolymerization rates (**a**) and monomers conversions (**b**) of the base composition (reference sample) and the compositions with the additional comonomer.

The research carried out in this study consisted of introducing an additional comonomer to the base monomers’ composition (BA, GMA, HEA) and determining its influence on the course of the bulk photopolymerization process and the physico-chemical properties of the obtained EARs. In the first stage, the kinetics of the photopolymerization process of the base monomer composition of BA/GMA/HEA (reference sample) and compositions with methacrylic or vinyl comonomer (MMA; EMA; BMA; LMA; AEM; NVP or STY) was monitored using photo-DSC. Each composition of monomers (as in Table 2) contained the same amount of photoinitiator TPO (0.01 mole). The results of these kinetics studies are shown in Figure 3.

As can be seen, systems with NVP and AEM exhibited a higher maximum photopolymerization rate (R_p_^max^) than the reference sample (>0.0017 s^−1^). An exceptionally high R_p_^max^ value was recorded for the sample with NVP (0.0034 s^−1^). It is known that NVP reduces the oxygen inhibition of acrylic monomers/oligomers photopolymerization, probably due to photo-oxidation or formation exiplex between oxygen and NVP [28]. As such, the photopolymerization process of BA/GMA/HEA in the presence of NVP is less oxygen sensitive and runs faster. The chemical structure of NVP also influences the photopolymerization rate. An NVP molecule has the carbonyl group and nitrogen molecule. In the case of polymerization of multifunctional monomers containing heteroatoms (like nitrogen, oxygen), the result of accelerating the process is known [29]. In systems with methacrylic monomer AEM, the R_p_^max^ value was also higher than in the reference sample (0.0024 s^−1^). AEM has two carbonyl groups in chain. In general, the introduction of a heteroatom into the monomer structure increases its reactivity in the photopolymerization process and increases its conversion. The other tested methacrylic monomers (MMA, EMA, BMA, and LMA) differ only in the number of carbon atoms in the aliphatic chain. With the increase in the length of the aliphatic chain in the methacrylate structure (from 1 to 4 carbon atoms), the R_p_^max^ values decreased (the reaction is slower), but at 11 carbon atoms (LMA), a similar R_p_^max^ value to the reference sample was recorded. The effect of lowering the polymerization rate with the length of the aliphatic chain in methacrylates has not yet been reported. This effect is also opposite to the cross-linking photopolymerization of multifunctional methacrylates with different chain lengths [29]. It is worth noting that the photopolymerization process of the system with styrene was completely different to the others. Not only was the photopolymerization rate very low from the beginning, but it was also almost constant over time (a slight decrease without a clear initial peak). Styrene is a particularly strong quencher of the excited states of the photoinitiator, resulting in the loss of the ability of the photoinitiator molecules to produce radicals. Therefore, acylphosphine oxide type photoinitiators are more dedicated to systems with styrene (they have a very short triplet lifetime).

The results of the conversion of double bonds (which, in the case of linear photopolymerization, can be identified with the monomers conversion) typically correlate with the results of the reaction rate. Namely, the higher the reaction rate of a given system, the higher the conversion of monomers (Figure 3b). Monomers conversion in reference sample was ca. 18%. The highest result of final conversion was recorded for the composition with NVP (48%). Significantly lower conversion was obtained for compositions with EMA (11%) and BMA (10%). Once again, the curve on the bottom of the chart came from the system containing styrene. The maximum conversion achieved was less than 3%. It is worth highlighting that the same amount of TPO photoinitiator (0.01 mole) was used in the photo-DSC tests. It should be noted that the additional methacrylic (MMA, EMA, BMA, LMA, AEM) and vinyl (NVP, STY) comonomers were introduced to base monomers compositions (BA, GMA, HEA) in an attempt to gain a higher level of control over the course of the FRBP process. Therefore, after analyzing the results obtained with photo-DSC, we decided to exclude NVP and AEM from further research because their addition causes a significant increase in the photopolymerization rate.

### 3.2. The Physicochemical Properties of the Epoxy Acrylic Resins

**Figure 4:**
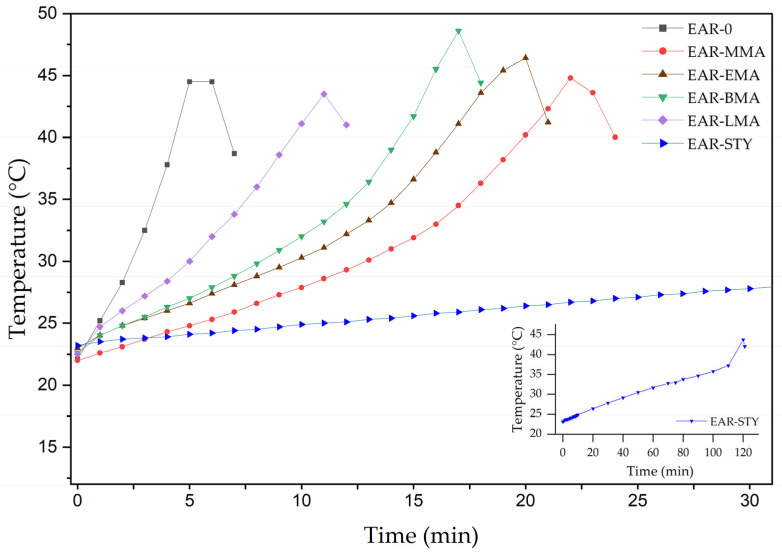
Temperature during the bulk photopolymerization process in glass reactor.

In the next stage of the research, a series of free radical photopolymerizations in bulk were carried out in a glass reactor. Epoxy acrylic resins (EARs) in the form of polymer syrups (solutions of terpolymer/tetrapolymers in unreacted monomers) were prepared. The monomers compositions, together with the used amounts of photoinitiator (TPO), are presented in Table 2. The content of TPO were selected experimentally, separately for each composition, so that during the synthesis its entire amount was decomposed. It was also important to obtain the highest possible conversion of the monomers, with a maximum viscosity of the syrups (ca. 10 Pa·s). Based on the data in Table 2, it can be seen that the system with STY required the largest amount of photoinitiator (0.24 mol), the EAR-LMA system had twice as much photoinitiator as the reference sample, EAR-BMA and EAR-EMA requires ca. 0.03 mole of TPO and EAR-MMA 0.04 mole of TPO. The course of the process was monitored by recording the temperature of the reaction mixture during the exposure to UV radiation. Processes were realized at an initial temperature of 21 ± 2 °C. The thermographs for the systems with different comonomers are presented in Figure 4.

As can be seen, the introduced additional comonomers clearly had an impact on the course of the photopolymerization process. EAR-0 reached a temperature peak (45 °C) after about 5 min of UV-irradiation. The introduction of the additional comonomer (methacrylic or vinyl) allowed the extension of the reaction time by at least 5 min with LMA up to 115 min with STY. Considering that the temperature increase during the reaction was similar for all syntheses (averaged 22 ± 2 °C), and the addition of mentioned comonomers extended the time to reach the temperature peak from approx. 100% (EAR-LMA) to over 400% (EAR-MMA), it can be said that their influence made the process more controllable. Styrene was unrivaled in terms of its influence on process control. The addition of this monomer allowed for an almost linear increase in temperature during the 120-min synthesis. This result was very similar to the data obtained earlier from photo-DSC. It was noticed that, in the case of methacrylic comonomers differing in the length of the aliphatic chain (MMA < EMA < BMA < LMA), the shorter the aliphatic chain of the monomer, the longer the time to reach the exothermic peak of the reaction. It can therefore be concluded that the reaction is milder. These results do not correlate with the photopolymerization rate determined by the photo-DSC method because the addition of photoinitiator differs (in DSC tests, the same amount of photoinitiator was used for all systems, while FRBP in the glass reactor shows the results for different content of photoinitiator), as well as the effect of mechanical mixing of comonomers (thin-layer photopolymerization in DSC) and process temperature control (isothermal tests in DSC).

The physico-chemical features of the EARs (i.e., dynamic viscosity, monomers conversions, solids contents, number and weight average molecular weights and polydispersity index) are presented in Table 3.

**Figure 5:**
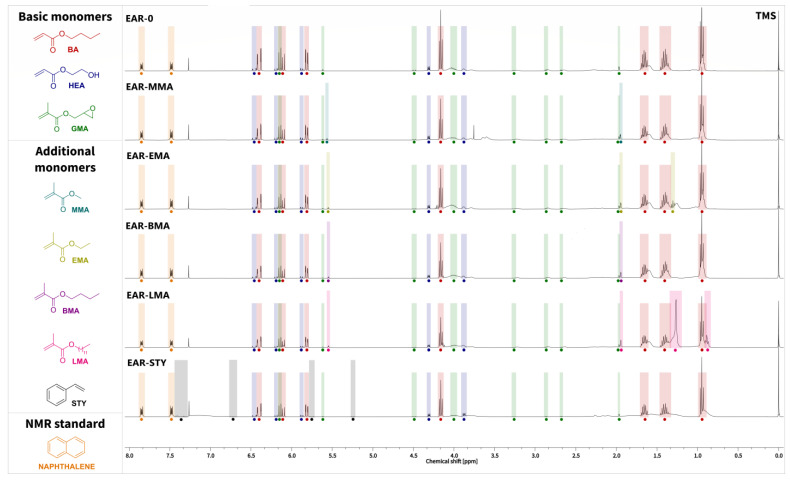
Stacked plot of the proton nuclear magnetic resonance (^1^H NMR) spectra of epoxy acrylic resins. Peaks monitored for each monomer and the internal standard (naphthalene) are indicated.

The monomers conversions values, determined based on NMR spectra (Figure 5), are very similar to the solids contents (SC), the values of which were determined by physical evaporation of unreacted monomers from resins (thermogravimetric method). However, the SC values were ca. 1–4% lower. This was probably caused by the fact that a small fraction of the monomers evaporated from the EAR sample during the sample’s preparation. Monomers conversion in EAR-0 reached the lowest value (40%). Attempts to achieve higher conversion by introducing more TPO photoinitiator, even by 0.001 mole (10% more), ended in gelation of the charge. It was therefore assumed that for this monomers-photoinitiator system, and under the used photopolymerization conditions, a conversion of approximately 40% is the maximum that can be achieved while maintaining the required product viscosity. As it turned out, the extension of the basic reaction mixture by the additional comonomer (methacrylic or vinyl) made it possible to obtain a higher monomers conversion. Adding methacrylate with a relatively short aliphatic carbon chain (MMA, EMA, and BMA) resulted in a significant increase in monomers conversion (approx. 60%) and useful viscosity (>10 Pa·s). It is significant that the highest monomers conversion was achieved by the sample containing styrene (67%), but that the viscosity of the EAR-STY syrup was only 3 Pa·s (similar as EAR-0).

The obtained terpolymer (BA-co-GMA-co-HEA) was characterized by similar values of molecular weights and polydispersity as tetrapolymer (BA-co-GMA-co-HEA-co-LMA). Already, photo-DSC studies have revealed that LMA does not affect the reactivity of BA/GMA/HEA systems (systems with similar photopolymerization rates). However, the addition of LMA allows for higher monomers conversion (i.e., lower VOC content). In turn, in the case of tetrapolymers with the participation of MMA, EMA or BMA, similar results of average molecular weights were obtained, but they were two times lower than for terpolymer. Once again, the tetrapolymer BA-co-GMA-co-HEA-co-STY stood out; Mn was only 2780 g/mol, Mw was 6,110 g/mol, and PDI value was the lowest at only 2.2. The formation of much shorter tetrapolymer chains made it possible to achieve the highest monomers conversion (67%) and the lowest viscosity (3 Pa∙s) of all obtained syrups.

The monomers’ conversions (total conversion and conversion of individual monomers) are presented in Table 4.

Considering the structures of the obtained epoxy acrylate polymers in syrups, it can be said that in both the case of EAR-0 containing the terpolymer BA-co-GEMA-co-HEA and in the case of the other EARs containing tetrapolymers, all types of unreacted comonomers were present in the syrups, and they mainly contain BA (ca. 60% in EAR-0 or EAR-LMA, ca. 50% in EAR with MMA, EMA or BMA, and only 40% in EAR-STY). Butyl methacrylate was used in the FRBP in the largest molar fraction. It is interesting that almost all the GMA amount is found in the terpolymer or tetrapolymer structures (more GMA is in tetrapolymer, 79–87%), but that an average of 20% unreacted GMA remains in the syrups. The same applies to the consumption of the additional (fourth) comonomer. Most (ca.80%) are in the structure of tetrapolymers, but styrene reacts as much as 95%. Thus, the epoxy acrylate resins obtained after the FRBP process consist of appropriate terpolymers or tetrapolymers and unreacted monomers, mainly BA and HEA (60 to 40%), and smaller amounts of GMA (20%) and additional comonomer.

### 3.3. Properties of UV-Crosslinked SATs Based on EARs

**Figure 6:**
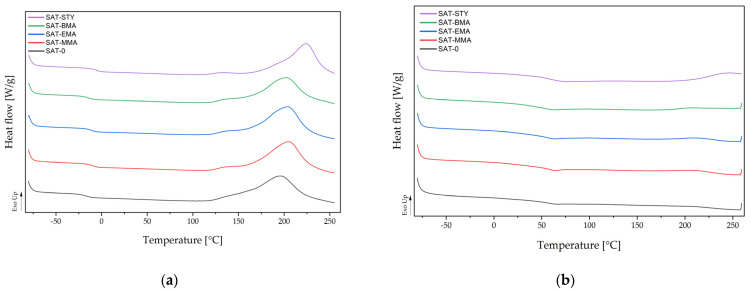
DSC thermographs of the UV-crosslinked SATs (**a**) and SATs after UV-crosslinking and thermal curing (**b**).

The obtained epoxy acrylic resins were used together with commercial epoxy resin (ER) and other additives to compound structural adhesive formulations allowing the production of thermally curable self-adhesive structural tapes (SATs), according to the methodology shown in Figure 2. The layer of the liquid composition (adhesive formulation) was transformed into a sticky solid with self-adhesive properties by means of UV radiation. After the UV-crosslinking process, it was revealed that the EAR-LMA is not compatible with the used epoxy resin. The produced adhesive film was white in color, very plasticky and greasy. For this reason, EAR-LMA was excluded from further research. The chemical structure of the obtained tetrapolymers (with hanging aliphatic chains of various lengths or with hanging benzene rings), as well as the content of linear tetrapolymers in EARs (identical to the SC values and monomers conversion), as well as the resulting content of cross-linked polyacrylate networks (created during UV-crosslinking process of SATs), determine the course of the thermal curing process of SATs (cationic polymerization of epoxy groups in the system derived from free GMA monomer in the syrups, epoxy groups from terpolymer or tetrapolymer structure and epoxy resin). Thermal analysis of UV-crosslinked SATs is presented in Figure 6 and Table 5.

As can be seen, the UV-crosslinked SATs (before thermal curing) exhibited one *T_g_* value. This suggested that the EARs and ER were thermodynamically miscible. As expected, the *T_g_* values differ due to the different composition of the polymer syrups and the significant differences in the glass transition temperatures of the homopolymers of the additional comonomers. SAT-0 (containing 8 moles of BA) was characterized by lowest *T_g_* value (−17 °C). Additional comonomers (MMA, EMA, BMA, or STY) were introduced into the monomer’s composition in place of the 2 moles of BA. These monomers are characterized by a higher *T_g_* of homopolymers than BA (*T_g_* of poly(BA) is −53 °C) [30], therefore the *T_g_* of SATs based on tetrapolymers increased slightly. This difference was particularly evident in the case of SAT-MMA, i.e., *T_g_* about —8 °C (*T_g_* of poly(MMA) is −105 °C) and SAT-STY, i.e., *T_g_* about −5 °C (*T_g_* of polystyrene is—100 °C). The difference in structure between the tetrapolymers and the terpolymer also affected the thermal hardening behavior of the SATs. The onset temperature of curing process (*T_i_*) for SAT based on EAR with tetrapolymers was much higher than for SAT-0 with the participation of terpolymer (ca. 20 °C). The exception was the system with styrene, for which the *T_i_* value reached 189 °C. A slightly increased maximum temperature of the curing process (+10 °C for SAT with methacrylic monomers, and +30 °C for SAT with STY) was also revealed. However, the enthalpy of the curing process was lower than for the reference sample. It can be concluded that the presence of larger structures in epoxy acrylate polymer chains (methyl group from methacrylates and an aromatic ring from STY) leads to steric obstacles, which likely reduces the enthalpy and increases the required curing temperatures for these SATs. This is also confirmed by the lowest value of crosslinking degree (0.92 a.u.) in case of SAT-STY. In the case of SATs with methacrylates, the α values were similar (0.97 or 0.96 a.u.) as for reference sample.

**Figure 7:**
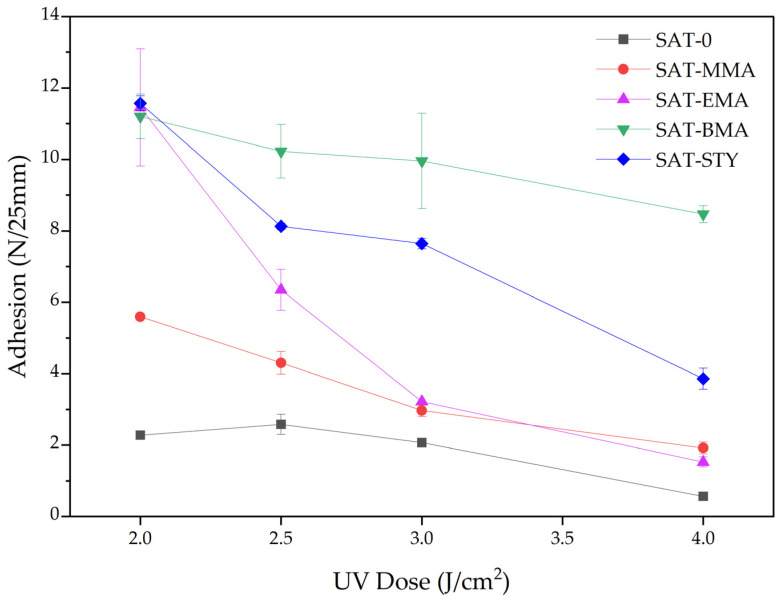
Adhesion to steel of UV-crosslinked SATs.

**Figure 8:**
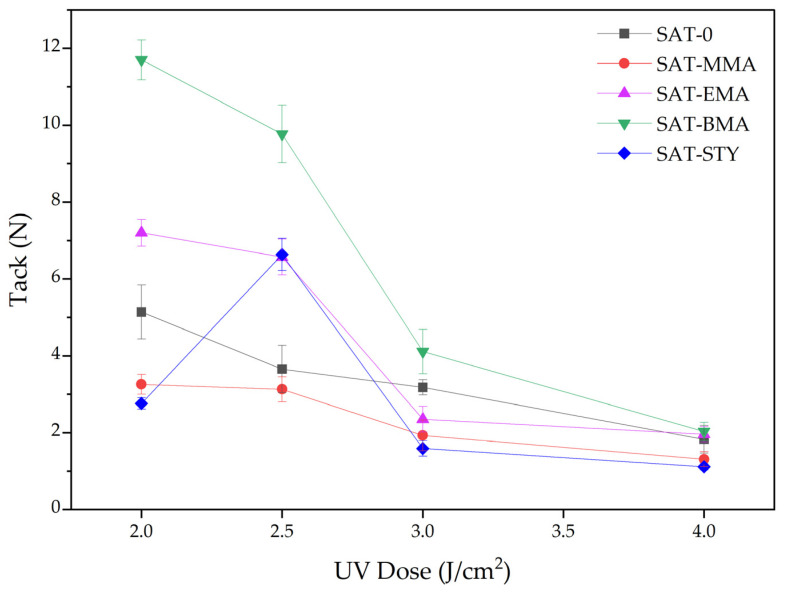
Tack to steel of UV-crosslinked SATs.

The UV-crosslinked SATs in a form of self-adhesive films (thickness of 120 ± 10 µm) were also characterized in terms of their basic self-adhesive features (i.e., adhesion to steel and tack). Results are presented in Figure 7 and Figure 8.

As can be seen, the values of adhesion and tack generally decrease with a higher UV-dose during crosslinking. Such a trend is consistent with other literature reports [31,32]. Several factors influence the examined properties: average molecular weights, polydispersity, the chemical structures of linear tetrapolymers and their content, as well as density of polymer network created from unreacted monomers (from EARs) and mulitunctional monomer during UV-crosslinking process of SAT films (Figure 2).

It should be mentioned that SAT-0 was obtained from EAR-0 syrup, in which the content of unreacted monomers was high (ca. 60%) and terpolymer BA-co-GMA-co-HEA characterized the highest average molecular weights, polydispersity. Thus, in this system there was relatively little linear terpolymer (containing no steric hindrances) and a dense polyacrylate network was formed (high content of unreacted monomers and multifunctional monomer). For this reason, the SAT-0 adhesion reached the lowest values (2.6 N/25 mm or less). The effect of lower adhesion for high molecular weight polymers [33,34] and dense polymer network structure in adhesive films are known [35]. In case of the SATs based on tetrapolymers, higher values of adhesion to steal and tack were generally achieved by systems with a longer aliphatic chain, i.e., SAT-BMA (11.2 N/25 mm and 11.7 N; respectively). These results correlate with the lower *T_g_* value for this tape (−14 °C). Interesting adhesion results were obtained for SAT-STY (from 11.8 N/25 mm to 4.2 N/25 mm). SAT-STY had the highest *T_g_* value (−5 °C). This is due to the high content of the linear tetrapolymer BA-co-GMA-co-HEA-co-STY (monomer conversion 67%) and its low molecular weight (Mn is 2700 g/mol; Mw is 6100 g/mol). During the investigation, no damage to the adhesive was observed.

### 3.4. Mechanical Properties of Thermally Cured SATs Based on Epoxy Acrylic Resins

**Figure 9:**
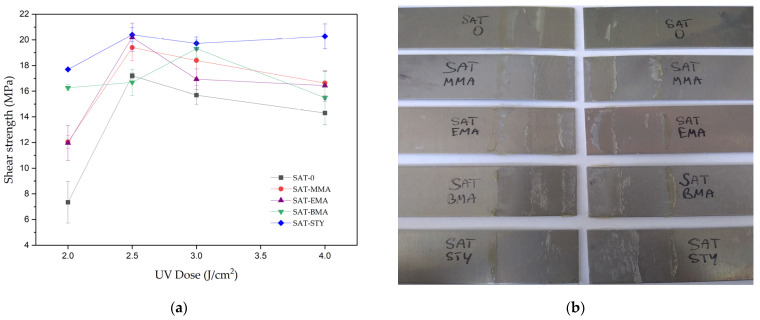
Shear strength of thermally cured aluminum–SAT–aluminum overlap joints (**a**) and view of aluminum panels after shear strength test (**b**).

The UV-crosslinked SATs were applied onto degreased 2024 T3 aluminum panels and the prepared Al/SAT/Al overlap joints were thermally cured at 170 °C for 60 min. Shear strength (*τ*) values recorded for the cured joints are presented in Figure 9.

SAT-0 based on terpolymer BA-co-GMA-co-HEA (the lowest content of linear polymer; 40%) generally showed the lowest shear strength values when compared to SATs based on tetrapolymers. The highest results of shear strength were obtained for joints with SAT-STY (17–20.8 MPa). Generally, for all Al/SAT/Al joints the maximum shear strength values were observed in the case of 2.5 J/cm^2^ of UV-dose (>16 MPa). An exception was SAT-BMA; the highest *τ* value reached 19.3 MPa (3 J/cm^2^ of UV-dose). Shear strength depends on the cross-linking degree of thermally cured SAT (*α*), which in turn is influenced by, among others, the length of the polymer chains (average molecular weights). Therefore, the highest shear strength was recorded for SAT-STY, characterized by the lowest α (0.92 a.u.) and molecular weights (Mn only 2 780 g/mol). Nevertheless, very high α values were achieved for all the SATs (>7 MPa). As presented in [24], a excessive *α* value may deteriorate the shear strength of Al/SAT/Al overlap joints. In addition, Figure 9b shows photos of adhesive joints after the shear test. In each case, the damage was of a mixed adhesive and cohesive nature, with adhesive damage predominating (approximately 80% in visual assessment), which confirms the high cohesion of the adhesive mass.

**Figure 10:**
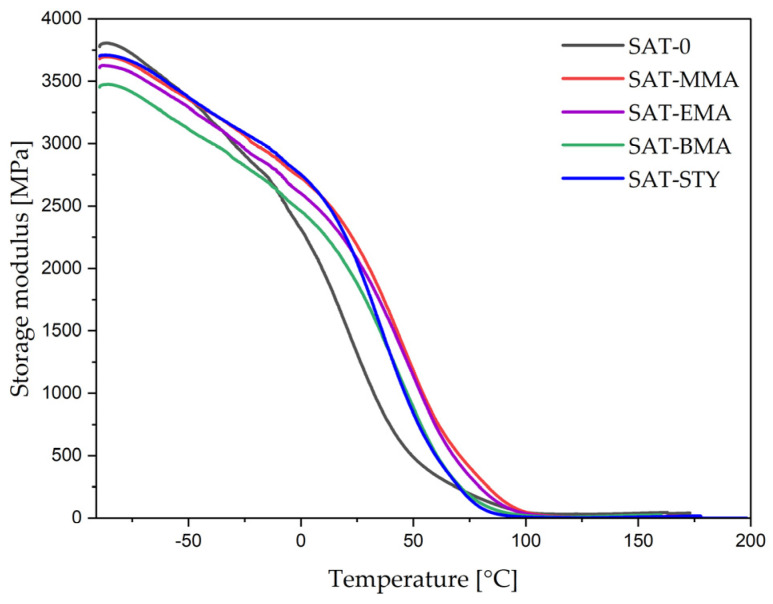
Storage modulus (E′) of thermally cured SATs.

**Figure 11:**
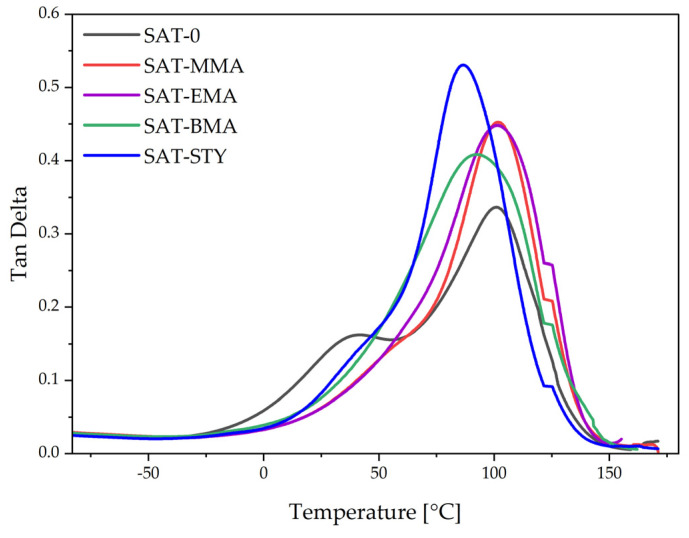
Tangens delta (tan δ) of thermally cured SATs.

The thermomechanical features of the thermally cured SATs were determined by the DMA technique (Figure 10 and Figure 11). The storage modulus and glass transition temperatures for cured SATs are summarized in Table 6. As can be seen, the storage modules at −50 °C for all SATs were similar and reached ca. 3300 MPa. Nevertheless, the glassy regime was significantly wider for SATs based on tetrapolymers than for SAT-0 based on terpolymer; the former materials were stiff up to room temperature. At higher temperatures (150 °C), higher storage modulus values were noted for SAT-0 and SAT-BMA (which achieved the highest *α*; 0.97 a.u.), and the lowest for SAT-STY (with the lowest α; 0.92 a.u.).

The DMA studies revealed that SAT-0 exhibits two glass transition temperatures: 30 and 102 °C. This likely resulted in phase separation of the epoxy components (the epoxy resin and polyacrylic network created during UV-crosslinking step from unreacted monomers). In the other systems, only one glass transition temperature was recorded for cured SATs. Higher *T_g_* values (approx. 100 °C) showed for SAT-MMA and SAT-EMA, and lower for SAT-BMA and SAT-STY. This is probably due to the higher proportion of linear tetrapolymers in EAR-BMA and EAR-STY (conversion of monomers was 62% and 67%, respectively; Table 4).

## 4. Conclusions

In this paper, the effect of methacrylic and vinyl comonomers (MMA, EMA, BMA, LMA, AEM, NVP and STY) on the course of the free radical bulk photopolymerization process (FRBP), the physico-chemical properties of obtained epoxy acrylic resins (EARs), as well as on thermal and mechanical features of structural adhesives are presented and disclosed. The main conclusions are as follows:

- Heteroatom-containing monomers (like NVP and AEM) are known to increase the rate of reaction and monomer conversion and should therefore not be used in FRBP;
- Methacrylic monomers with short aliphatic chain (C_1_–C_4_) allow the reduction of the rate of photopolymerization reaction, extends the time to reach the temperature peak and increases the conversion of monomers (up to ca. 60%). On the other hand, long-chain methacrylates (C_11_) do not significantly reduce the rate of photopolymerization, but slightly increase the conversion of monomers (about 15% compared to the reference sample). However, the resulting tetrapolymers are characterized by higher polydispersity;
- Styrene has the greatest impact on reducing the reaction rate and increasing monomer conversion (up to 67%). Significantly more photoinitiator should be used for the photopolymerization process involving styrene. The resulting tetrapolymers have very low average molecular weights. Additionally, structural adhesives based on epoxy acrylate resin with STY were characterized by high adhesion to steel (9–11.5 N/25 mm) and shear strength (20.8 MPa).

## Figures and Tables

**Table 1:** Chemical structures of used comonomers.

Base Monomers			
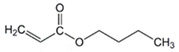 Butyl acrylate (BA)	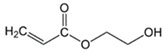 2-Hydroxyethyl acrylate (HEA)		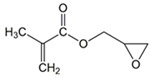 Glycidyl methacrylate (GMA)
Methacrylic comonomers			
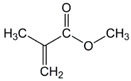 Methyl methacrylate (MMA)	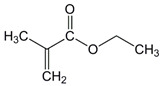 Ethyl methacrylate (EMA)		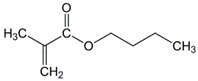 Butyl methacrylate (BMA)
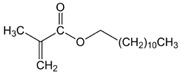 Lauryl methacrylate (LMA)		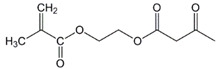 (2-Acetoacetoxy)ethyl methacrylate (AEM)	
Vinyl comonomers			
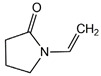 N-vinylpyrrolidone (NVP)		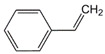 Styrene (STY)	

**Table 2:** Compositions of monomers and photoinitiator for preparation of epoxy acrylic resins.

EAR Symbol	Monomers (mol %)				TPO *
	Base Monomers			Additional Monomer (20 mol %)	(mol)
	BA	HEA	GMA		
EAR-0	80	10	10	-	0.01
EAR-MMA	60	10	10	MMA	0.04
EAR-EMA				EMA	0.035
EAR-BMA				BMA	0.032
EAR-LMA				LMA	0.022
EAR-AEM				AEM	—
EAR-NVP				NVP	—
EAR-STY				STY	0.24

* Amount of TPO used during photopolymerization in glass reactor (not in photo-DSC tests); —n.d.

**Table 3:** Monomers conversion according to NMR data, solids content, dynamic viscosity, average molecular weights and polydispersity index of EARs.

EAR Symbol	Monomers Conversion (%)	SC (%)	η (Pa∙s)	*M*_n_ (g/mol)	*M*_w_ (g/mol)	PDI
EAR-0	40	37	3.5	21,410	82,120	3.83
EAR-MMA	60	56	8	10,550	32,120	3.04
EAR-EMA	59	58	10	11,490	38,290	3.33
EAR-BMA	62	59	11	11,790	30,570	3.27
EAR-LMA	56	54	6	21,770	85,090	3.9
EAR-STY	67	64	3	2780	6110	2.2

**Table 4:** Monomers’ conversion calculated by proton nuclear magnetic resonance (^1^H NMR) analyses.

EAR Symbol	Monomers Conversion (%)	Conversion of Individual Comonomers (%)							
		BA	GMA	HEA	MMA	EMA	BMA	LMA	STY
EAR-0	40	36	73	37	-	-	-	-	-
EAR-MMA	60	50	84	48	87	-	-	-	-
EAR-EMA	59	48	84	49	-	85	-	-	-
EAR-BMA	62	51	86	52	-	-	84	-	-
EAR-LMA	56	42	79	45	-	-	-	74	-
EAR-STY	67	58	87	57	-	-	-	-	95

**Table 5:** Thermal features of UV-crosslinked SATs (UV dose of 3 J/cm^2^) and cross-linking degree of thermally cured SATs.

SAT Acronym	*T_g_* (°C)	*T_i_* (°C)	*T_p_* (°C)	Δ*H* (J/g)	*α* (a.u.)
SAT-0	−17	147	196	247	0.97
SAT-MMA	−8	160	206	214	0.96
SAT-EMA	−11	161	205	215	0.96
SAT-BMA	−14	160	202	194	0.97
SAT-STY	−5	189	225	183	0.92

*T_g_* is the glass transition temperature; *T_i_* is the onset temperature of the curing reactions; *T_p_* is maximum temperature of the curing reaction; Δ*H* is the enthalpy of SAT curing processes; *α* is the cross-linking degree of thermally cured SATs.

**Table 6:** Storage modulus (E′) and the glass transition temperature (*T_g_*) of cured SATs.

SAT	*E*′ _(−50 °C)_ (MPa)	*E*′ _(25 °C)_ (MPa)	*E*′ _(150 °C)_ (MPa)	*T_g_* (°C)
SAT-0	3 358	1 310	38	30 and 102
SAT-MMA	3 350	2 165	10	101
SAT-EMA	3 285	2 070	15	100
SAT-BMA	3 112	1 875	21	92
SAT-STY	3 365	2 050	9	86

## Data Availability

The data presented in this study are available on request from the corresponding author.
